# Typology of out-of-home eaters: a description of sociodemographic, lifestyle, nutritional and environmental characteristics in the NutriNet-Santé cohort

**DOI:** 10.1186/s12966-025-01752-5

**Published:** 2025-05-26

**Authors:** Emma Meyer, Benjamin Allès, Justine Berlivet, Sandrine Péneau, Alice Bellicha, Mathilde Touvier, Brigitte Langevin, Philippe Pointereau, Denis Lairon, Serge Hercberg, Emmanuelle Kesse-Guyot, Julia Baudry

**Affiliations:** 1 Center of Research in Epidemiology and StatisticS (CRESS), Nutritional Epidemiology Research Team (EREN), Université Sorbonne Paris Nord and Université Paris Cité, Inserm, INRAE, CNAM, Bobigny, F-93017 France; 2 Solagro, 75 Voie TOEC, Toulouse Cedex 3, F-31076 France; 3 Aix Marseille Université, Inserm, INRAE, C2VN, Marseille, 13005 France

**Keywords:** Eating out of home, Diet quality, Dietary behaviours, Diet sustainability, Observational study

## Abstract

**Background:**

The out-of-home (OOH) food sector holds the potential to promote healthier and more sustainable diets on a large scale given the high number of people eating OOH regularly. However, information about socioeconomic and dietary characteristics of OOH eaters is limited. This study aimed to identify a typology of OOH consumers by frequency and type of meal consumed OOH and their associated sociodemographic, lifestyle, nutritional, and environmental characteristics.

**Methods:**

Based on a sub-sample of adults of the French NutriNet-Santé cohort who completed a food frequency questionnaire and a questionnaire on OOH consumption habits in 2014 (*n* = 29,140, mean age: 53.6 (SD = 14.0) years, 74.3% women), we conducted a Multiple Factor Analysis followed by a clustering procedure. AN(C)OVA models were then used to examine the associations between the identified clusters and socio-demographic, lifestyle, and diet-related characteristics (using dietary scores and environmental indicators including greenhouse gas emissions, land use and energy consumption).

**Results:**

We identified five clusters based on their OOH consumption patterns: *Weekday-only eaters* (19%), *Frequent weekday and weekend eaters* (24%), *Organic eaters* (6%), *Weekend and evening eaters* (19%), and *Home-only eaters* (32%). *Weekday-only eaters* were younger, more likely to be professionally active and to have children at home than the other groups. *Frequent weekday and weekend eaters*, with the highest OOH consumption, had the lowest dietary quality and the highest diet-related environmental impacts and consisted mostly of younger women with higher socioeconomic status. *Organic eaters*, often living in urban areas and following specific diets such as vegan or vegetarian ones, had the best dietary quality and the lowest diet-related environmental impacts. *Weekend and evening eaters* and *Home-only eaters* had a higher proportion of retired individuals, with *Weekend and evening eaters* also showing a greater proportion of high-income individuals.

**Conclusions:**

Our findings indicate a lower dietary quality and higher dietary environmental impacts among frequent OOH eaters, whereas those with higher organic consumption showed opposite trends. This study contributes to the understanding of different OOH consumer characteristics and could provide a basis for further research in the field.

**Trial registration:**

The study was registered at ClinicalTrials.gov (NCT03335644).

**Supplementary Information:**

The online version contains supplementary material available at 10.1186/s12966-025-01752-5.

## Background

To address public health and environmental challenges of current food systems, effective and targeted large-scale strategies are needed to promote sustainable diets, i.e., socially and economically acceptable diets with low environmental impacts and high nutritional quality [1]. In this context, the out-of-home (OOH) sector may be a promising lever towards healthier and more environmentally sustainable diets, given the high number of individuals eating OOH regularly [2]. In Western countries, OOH meals account for a significant portion of dietary intake, with 13% of meals in France consumed in OOH settings [3] and 30% of working or studying people having lunch at their workplace [4]. The definition of eating OOH varies among studies [5]. Generally, OOH consumption refers to any food that is not both prepared and consumed at home [3, 6], such as meals from restaurants, cafés, take-aways, or community catering in schools, workplaces, hospitals or care facilities.

Existing research on OOH consumption primarily focuses on the nutritional dimension of dietary sustainability [5, 7, 8]. Previous reviews on OOH consumption, conducted in Western countries and throughout different populations, showed that a higher frequency of eating OOH was generally related to higher body weight and poorer diet quality [5, 7, 8]. In addition, they concluded that meals produced outside home contribute less to a healthy diet than home-made meals.

The environmental aspects of OOH meals have been less frequently assessed, though OOH facilities generate significant emissions, food waste, and resource use [9]. Life cycle assessments suggest a great potential to reduce environmental impacts of diets, especially by adapting the menu design [10–12]. While environmental and nutritional impacts of meals are often interrelated [13], there is limited understanding of how different OOH settings influence sustainable diets.

Focusing on OOH rather than at-home food preparation can have large-scale sustainability effects as OOH meals are prepared in large quantities and consumed by a vast number of individuals. Unlike home cooking, OOH consumers have limited control over meal composition, which makes the sustainability of the offering even more important.

Furthermore, studies [5, 7] found relevant sociodemographic differences in OOH consumers’ profiles. Individuals who frequently eat OOH generally have higher education, income, are younger, and more often male [5]. However, OOH consumption is complex, involving various settings and motivations, and hence requires a more detailed characterisation [5, 7].

While existing studies [5, 7] mostly focus on associations of eating OOH with dietary quality, little is known about detailed profiles of consumers eating OOH, including their OOH eating behaviour at different times of the week and different settings of consumption. In addition, no studies have simultaneously described sociodemographic, lifestyle as well as diet-related characteristics of different OOH eater profiles, in particular little attention has been paid to their diet-related environmental impacts.

In that context, the present work aimed to contribute to evidence by characterising OOH consumers’ diets to inform targeted strategies for healthier and more sustainable OOH consumption. More specifically, we aimed to (1) identify a typology of consumers eating OOH and their associated sociodemographic and lifestyle characteristics and (2) describe the typology’s diets using a multicriteria approach covering various indicators related to nutrition, environment, and economy.

## Methods

### Study population

The current study is a cross-sectional analysis using data from the French NutriNet-Santé study collected in 2014. The NutriNet-Santé study, which has been described in detail previously [14], is an ongoing web-cohort (www.etude-nutrinet-sante.fr) launched in 2009 with the objective to assess the links between health and nutrition. Recruitment was initially launched through a nationwide multimedia campaign and is still open, allowing all adults from the general population with access to the Internet to participate. Inclusion requires completing semi-annually or annually administered questionnaires on sociodemographics and anthropometrics, dietary intake, lifestyle, and physical activity. Throughout follow-up, detailed questionnaires on other relevant topics are completed.

### Data collection

#### Assessment and definition of OOH consumption

Data on OOH consumption behaviour was obtained through the previously described questionnaire [15, 16] about eating practices, attitudes and motivations when purchasing food, administered to NutriNet-Santé participants from July until December 2014 [17].

This questionnaire collected data on the absolute number of eating OOH for the following three occasions: “lunchtime on weekdays”, “evening on weekdays” and “weekends”. For weekdays, participants reported how many of 5 lunches and 5 dinners were eaten OOH on scales from 0 to 5. For weekends, the total number was reported on a scale from 0 to 4 (comprising lunch and dinner). Furthermore, for “lunchtime on weekdays” and “evening on weekdays and weekends” questions were asked about the frequency of meal types consumed when eating OOH, distinguishing between restaurant, delivery service, take-away, self-prepared meals, canteen meals (generally comprised of starter, main course, dessert) or eating at family/friends. For the first four meal types, a further distinction was made between organic and non-organic options, given the increasing importance of organic food in the OOH market (e.g. the French EGalim regulation aims for 20% organic food in collective catering [18]). Organic refers to products certified under the European Union regulation (EC) No. 834/2007. A 5-modality scale was used to assess the frequency per type of meal (“never”, “rarely”, “from time to time”, “often”, “always”). Individuals who stated that they did not eat OOH at one or more occasions were given the values “never” for the frequency of type of meals and “0” for the absolute number of eating OOH at the respective occasion.

Since there is no standardised definition of OOH food, in this study, OOH consumption was defined based on the settings covered by the aforementioned questionnaire. This included meals prepared at home but consumed away from home as well as food bought outside the home but possibly consumed at home (such as delivery service or take-away).

#### Sociodemographic and lifestyle data

Sociodemographic data was collected at participants’ inclusion and every year thereafter using a self-administered questionnaire including information on sex, age, occupation, education, residence area, income, marital status, and presence of children in the household [19, 20]. Income was calculated per household unit, where the first adult was defined as 1, other members of the household over ≥ 14 years were defined as 0.5 and children under the age of 14 as 0.3, based on the definition of the National Institute of Statistics and Economic Studies (INSEE) [21].

Physical activity was collected using the International Physical Activity Questionnaire [22]. Based on the Metabolic Equivalent of Task (MET) minutes per week (MET-min/week), three levels of physical activity were defined: low (< 600 MET-min/week), moderate (600 to 1500 MET-min/week) and high (> 1500 MET-min/week). Smoking status was self-reported by participants’ according to the categories “smokes daily”, “smokes occasionally”, “former smoker” and “never smoker”. The first two categories were combined. Additionally, specific dietary behaviour was assessed using the food frequency questionnaire (FFQ) described below. Pescatarians were defined as participants not eating meat but eating fish. Vegetarians were defined as those neither eating meat nor seafood but eating eggs and dairy products, and those not eating any animal-based products were defined as vegans.

This data was regularly collected as part of the follow-up study, so data points closest to the completion of the questionnaire on eating practices, attitudes and motivations were used.

#### Dietary data

Dietary data was recorded through a self-administered organic FFQ (Org-FFQ) differentiating organic and non-organic food consumption. It was administered concomitantly with the aforementioned questionnaire on eating practices, attitudes and motivations and has been described elsewhere [23]. Briefly, the Org-FFQ, built upon on a validated FFQ [24], included 264 food items. For each item, participants estimated the consumed quantity (using validated coloured photographs or standard portion sizes) [25] and the frequency of consumption. In addition, organic consumption was administered for each food item on a 5-point-scale (“never”, “rarely”, “half of time”, “often”, “always”). The share of organic food in the diet was calculated by weighting the modalities (0, 0.25, 0.5, 0.75, 1) and dividing organic food intake by the total food intake (g/d) while excluding water.

Daily nutrient intakes were calculated using the NutriNet-Santé composition dataset [26] that is mainly based on the official French composition table Ciqual [27]. This dataset is maintained by a team of dietitians to ensure the use of validated data.

Various dietary quality scores were calculated. The PNNS-GS2 (Programme National Nutrition Santé-Guidelines Score 2) assesses adherence to French dietary guidelines, considering 13 components (7 beneficial and 6 unfavourable), with a range of -∞ to 14.25, based on the consumed quantities. Higher values represent a higher adherence to the national dietary guidelines [28]. The PANDiet (Diet Quality Index based on the Probability of Adequate Nutrient Intake) captures 28 nutrients whose intakes should meet a certain reference, along with six nutrients that should not exceed a certain reference. The score ranges between 0 and 100, with higher values indicating a better nutrient adequacy [29]. The computation of the cDQI (Comprehensive Diet Quality Index) is based on 11 plant-based foods and 6 animal-based foods. Points are attributed regarding the consumed quantity ranging from 0 to 5 points per component and resulting in a theoretical score range from 0 to 85. Higher scores represent a better dietary quality [30]. The ELD-I (EAT-Lancet Diet Index) reflects adherence to the EAT-Lancet recommendations, which promote plant-based foods to respect both planetary boundaries and human health [31]. The score is based on the consumption quantity of 14 food groups and results in continuous values, with higher values representing a higher adherence to the EAT-Lancet dietary recommendations [32].

More detailed information on the scores can be found in Supplemental Method 1, Additional File.

#### Environmental data

The environmental data includes GHGe, (kg CO_2_-eq), used land surface (LU, m^2^) and cumulative energy demand (CED, MJ) of the participants’ usual diet. Values were obtained using the DIALECTE tool [33] developed by Solagro, providing data from life cycle assessments up to the farm gate (i.e., post-farm processes such as transporting or packaging are not considered), while distinguishing between organic and conventional food production. The computation has been described in detail previously [34]. Environmental impacts per day per individual were calculated based on the participants’ consumption. For each indicator, higher values indicate higher environmental impacts.

#### Economic data

Data for diet monetary cost was obtained by merging food consumption data from the Org-FFQ and participants’ data on place of purchase with food prices of the 2012 Kantar Worldpanel database [35], which holds data on mean prices for organic and conventional food items.

### Statistical analysis

#### Selection of study sample

A total of 40,680 NutriNet-Santé volunteers had filled out the questionnaire on eating practices, attitudes and motivations, and had no missing values on the questions pertaining to OOH consumption. This data was merged with data of 29,210 individuals having completed the Org-FFQ with full information on all investigated indicators. Thus, the final sample consisted of 29,140 individuals. A flowchart is provided in Supplemental Fig. 1, Additional File.

#### Construction of eating OOH typology

The construction of clusters was based on the absolute number of times individuals ate OOH at lunchtime on weekdays, evening on weekdays, and on weekends (quantitative variables) and the frequency of type of meals consumed. The latter included questions on a 5-point-scale (“never”, “rarely”, “from time to time”, “often”, “always”) on the following types of meal: restaurant, delivery service, take-away, self-prepared meals, canteen meals or eating at family/friends that were available for two time points (“at lunchtime” and “evening on weekdays and weekends”), distinguishing between organic and non-organic options for the first four types. For the analysis, these categories were collapsed into three: “never”, “sometimes” (combining “rarely” and “from time to time”), and “frequently” (combining “often” and “always”). This resulted in 20 categorical variables, each with 3 modalities, and 3 quantitative variables.

A Multiple Factor Analysis (MFA) was conducted using these 23 variables which were divided into groups. The 20 variables on types of meals were considered as four groups, separating the variables at lunchtime on weekdays, and on weekday evenings and weekends, as well as organic and non-organic options. In view of the upcoming cluster analysis, no rotation method was applied. The first two dimensions explained 21.3% of the variance. Three dimensions (26.6% of the variance) were kept for the following clustering procedure based on the elbow method using the scree plot of variances per dimension. In addition, these dimensions had an eigenvalue of > 1. To determine the optimal number of clusters, the total within-cluster sum of squares (representing the intra-cluster variation) was computed for different cluster sizes. Using the elbow method, an optimal number of 5 clusters was detected. An Ascending Hierarchical Classification procedure using Ward’s method was conducted, which was stabilised by a K-means procedure. These analyses were performed using the “MFA” and “HCPC” functions of the “FactoMineR” package in R. The scree plots, as well as the factor loadings can be found in Supplemental Method 2, Additional File.

Given the possibility that individuals consuming self-prepared meals OOH may differ from other OOH eaters, we examined the contribution of these variables to the main MFA dimensions and re-ran the full analysis excluding them. As both approaches showed limited impact, all variables were retained.

#### Description and comparison of clusters

Clusters were named based on the variables that most distinctly defined each group. Specifically, the point of the week and the type of meal played a key role in the naming process. They were then described in terms of OOH consumption behaviour, sociodemographic, lifestyle, nutritional, economic, and environmental characteristics, using means and 95%-CI for quantitative and percentages for categorical variables, respectively.

Associations of nutritional, environmental, and economic indicators related to the overall dietary patterns across clusters were estimated using ANCOVA models adjusted for energy intake (Model 1) and for sex, age and energy intake (Model 2), respectively. P-values were reported using chi-square tests for categorical variables and type III AN(C)OVA tests for continuous variables.

Data management and statistical analyses were performed using RStudio software (RStudio, 2023.12.1 Build 402 © 2009–2024 Posit Software, PBC).

## Results

The full sample (*n* = 29,140) had an average age of 53.6 (SD = 14.0) years and consisted of 74.7% women. Five clusters were identified based on their OOH consumption behaviour representing 19% (*Weekday-only eaters*), 24% (Fr*equent weekday and weekend eaters*), 6% (*Organic eaters*), 19% (*Weekend and evening eaters*), and 32% (*Home-only eaters*) of the sample. The values of cluster the constructing variables can be found in Supplemental Table 1, Additional File.

**Fig. 1:**
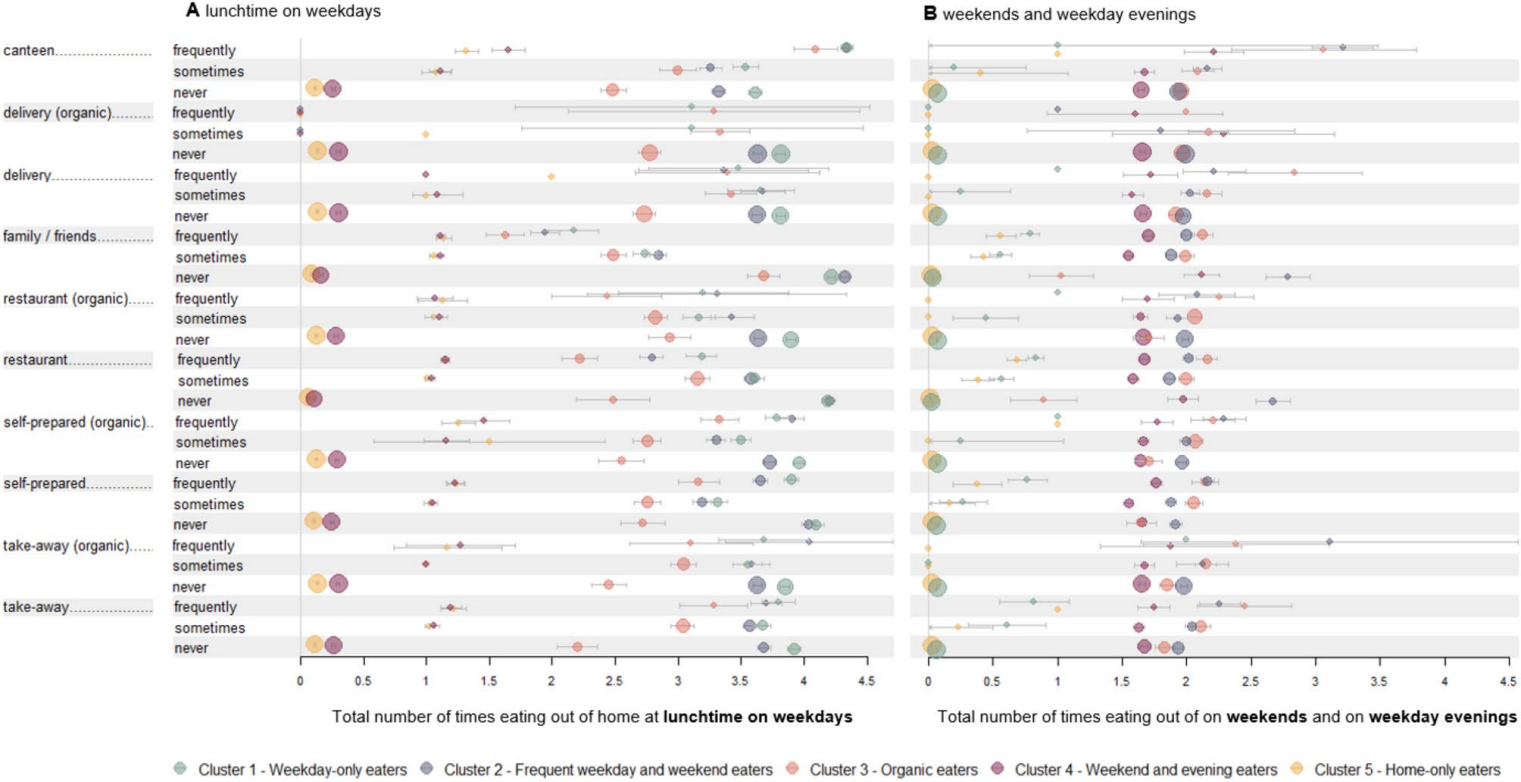
Out-of-home eating frequency by occasion, setting, and out-of-home eating behaviour-based clusters. (**A**: lunchtime on weekdays, **B**: weekends and weekday evenings). Circle sizes are proportional to the number of individuals in each cluster corresponding to the modalities. Bars indicate 95%CI, colours indicate the different clusters. *N* = 29,140 Reading note: For the category “canteen” in panel **A** (first three lines), Weekday-only eaters and frequent weekday and weekend eaters have a greater number of individuals eating frequently at the canteen than the other clusters. These individuals eat on average more than 4 times per week OOH at lunchtime on weekdays (line 1). The Organic eaters, Weekend and evening eaters and Home-only eaters have a smaller proportion of individuals eating frequently at the canteen. Conversely, The Weekend and evening eaters and Home-only eaters have a high proportion of individuals who never eat at the canteen and at the same time eat very little OOH in terms of absolute numbers (line 3)

The specific OOH behaviours (Fig. 1) and sociodemographic characteristics (Table 1) are described below.

**Table 1:** Sociodemographic and lifestyle indicators across out-of-home eating behaviour-based clusters, NutriNet-Santé study (2014, *n* = 29,140)^1^

		Whole sample	Cluster 1		Cluster 2	Cluster 3	Cluster 4	Cluster 5
			Weekday-only eaters		Frequent weekday and weekend eaters	Organic eaters	Weekend and evening eaters	Home-only eaters
*N*		*29*,*140*	*5598*		*6999*	*1820*	*5540*	*9183*
*%*		100.00	*19.21*		*24.02*	*6.25*	*19.01*	*31.51*
Sex (%)								
	Female	74.73	75.15		80.45	79.73	76.23	68.21
	Male	25.27	24.85		19.55	20.27	23.77	31.79
Age (years)								
	Mean	53.55	48.01		45.65	49.49	57.06	60.83
	SD	13.97	11.94		13.11	13.75	12.96	11.59
Educational attainment (%)								
	< High-school diploma	21.31	13.52		10.42	12.31	23.90	34.59
	High school diploma	14.64	13.20		11.89	10.88	15.65	17.74
	Postgraduate	64.05	73.28		77.70	76.81	60.45	47.68
Occupational status (%)								
	Unemployed	4.06	2.41		2.97	5.88	5.42	4.72
	Never employed	6.86	3.80		5.27	6.43	9.78	8.27
	Self-employed, farmer	1.75	1.32		1.30	2.58	2.17	1.93
	Employee, manual worker	14.30	22.03		17.03	13.13	12.27	8.96
	Intermediate professions^a^	14.71	24.88		24.13	15.27	8.45	4.99
	Managerial staff, intellectual profession	21.01	33.99		35.42	31.65	11.32	5.86
	Retired	37.31	11.56		13.87	25.05	50.60	65.28
Monthly income per household unit (%)^b^								
	Unwilling to answer	6.04	5.47		4.93	5.33	6.41	7.17
	<€1200	11.56	11.59		9.76	10.49	11.59	13.11
	€1200–1800	23.11	25.72		20.76	21.10	21.32	24.78
	€1800–2700	27.51	26.97		27.99	28.68	26.16	28.05
	>€2700	31.78	30.24		36.56	34.40	34.53	26.89
Place of residence (%)								
	Rural community	22.61	23.65		18.00	16.37	23.03	26.47
	Urban unit with a population < 20,000	15.33	15.31		12.07	10.55	16.39	18.12
	Urban unit with a population between 20,000 and 200,000 inhabitants	18.19	16.01		15.72	16.04	20.00	20.76
	Urban unit with a population > 200,000	43.87	45.03		54.21	57.03	40.58	34.65
Children (%)								
	Children at home	23.33	39.37		30.13	23.08	15.99	12.85
	No children at home	76.67	60.63		69.87	76.92	84.01	87.15
Marital status (%)								
	Single	12.54	14.20		18.89	18.85	9.68	7.15
	Married	56.55	53.72		43.32	40.16	61.77	68.45
	In a relationship (civil union, cohabitation)	17.78	21.54		25.92	25.88	14.01	9.96
	Divorced or separated	9.79	8.72		9.54	11.81	10.23	9.96
	Widow(er)	3.34	1.82		2.33	3.30	4.31	4.46
Smoking status (%)^c^								
	Never smoker	48.81	54.29		50.66	46.26	44.57	47.12
	Former smoker	40.45	35.35		34.78	39.29	44.46	45.70
	Current smoker	10.74	10.36		14.56	14.45	10.97	7.18
Physical activity (%)^d^								
	Missing	10.78	10.81		10.36	8.85	10.38	11.72
	Low	33.63	27.99		26.66	34.56	37.49	39.87
	Moderate	36.38	38.42		41.32	42.53	34.10	31.51
	High	19.21	22.78		21.66	14.07	18.03	16.90
Dietary behaviour (%)^e^								
	Omnivore	95.61	94.98		96.69	91.10	96.05	95.82
	Pescatarian	1.74	1.73		1.30	2.91	1.77	1.84
	Vegetarian	1.77	2.36		1.39	3.85	1.39	1.54
	Vegan	0.87	0.93		0.63	2.14	0.79	0.81

^a^ Intermediate professions include occupations that require specialised skills or education but do not require a university degree

^b^ Income was calculated per household unit as described in the Methods section

^c^ Smoking status was self-reported by participants using categories described in the Methods section

^d^ Physical activity was assessed using the using the International Physical Activity Questionnaire (IPAQ) as described in the Methods section

^e^ Dietary behaviour was captured using a Food Frequency Questionnaire (FFQ) as described in the Methods section

^1^ Values are means for continuous variables and percentages for categorical variables

P-values are based on type III ANOVA test or χ2 test, as appropriate, all P-values < 0.0001

### Sociodemographic and OOH consumption indicators

#### Weekday-only eaters (19%)

This group frequently ate OOH at lunchtime on weekdays (on average 3.8 times/week) but usually did not eat OOH on weekends or weekday evenings. Their preferred OOH meals were self-prepared dishes and canteen meals, or sometimes restaurant or take-away meals.

*Weekday-only eaters* had an average age of 48.0 (SD = 11.9) years and were 75.2% women, close to the average of the whole sample. This cluster had the highest proportion of individuals having children at home (39.4%) and the smallest proportion of unemployed (2.4%), never employed (3.8%) and retired (11.6%) individuals. Furthermore, the highest percentage of never smokers (54.3%) and individuals with a high physical activity level (22.8%) was found in this group.

#### Frequent weekday and weekend eaters (24%)

This group had the highest overall OOH frequency, averaging 3.6 times/week at lunchtime on weekdays and 2 times/week on weekday evenings and weekends. When they ate OOH at lunchtime, they often consumed self-prepared meals or ate at the canteen. Sometimes they ate at restaurants, at family/friends or consumed take-away foods. When eating OOH in the evenings or on weekends, they tended to eat self-prepared dishes, at family/friends or at restaurants.

*Frequent weekday and weekend eaters* were on average the youngest (45.7 years, SD = 13.1) and had the highest proportion of female participants (80.5%). This group contained the highest share of individuals with postgraduate education (77.7%), of individuals with a monthly income per household unit > 2700€ (36.6%), and singles (18.9%). More than half of the individuals (54.2%) of this cluster lived in urban areas of more than 200,000 inhabitants. Similar to *Weekday-only eaters*, this group had a higher percentage of individuals with high physical activity than the average sample (21.7%).

#### Organic eaters (6%)

This group had the second highest overall frequency of eating OOH. When they ate OOH at lunchtime on weekdays (on average 2.8 times/week) they primarily ate self-prepared meals or sometimes at restaurants but rarely at the canteen. On weekends and weekday evenings (on average 2 times/week), they additionally ate more often at family/friends.

*Organic eaters* had an average age of 49.5 (SD = 13.8) years and consisted of 79.7% women, higher than the average of the whole sample. A high number of individuals in this cluster had a post-graduate diploma (76.8%). Furthermore, the highest share of self-employed individuals (2.6%) and individuals living in urban areas with a population of > 200,000 (57%) was found in this cluster. In addition, this group displayed the highest proportions of vegans, vegetarians or pescatarians.

#### Weekend and evening eaters (19%)

This cluster usually did not eat OOH at lunchtime on weekdays but at weekends or on weekday evenings (on average 1.7 times/week). When they ate OOH, they usually ate self-prepared dishes, at restaurants or at their family/friends.

*The Weekend and evening eaters* were on average 57.1 (SD = 13.0) years old and included 76.2% women, close to the whole sample’s average. This cluster had the second highest age and the highest proportion of never employed individuals (9.8%).

#### *Home-only eaters* (32%)

This cluster was composed of subjects who usually did not eat OOH.

*Home-only eaters* were characterised by the highest average age (60.8 years, SD = 11.6), the highest proportion of men (31.8%), individuals without a high-school diploma (34.6%), retirees (65.3%), and residents of rural areas (26.5%). This cluster also had the highest percentage of married individuals (68.5%) and those with low physical activity levels (39.9%), as well as the lowest proportion of current smokers (7.2%). Additionally, only 26.9% of individuals in this group had a monthly household income exceeding €2700.

### Nutritional and cost indicators across clusters

After adjustment for energy intake (model 1), *Organic eaters* had the highest dietary scores (PNNS-GS2, PANDiet, ELD-I, cDQI). As these indices capture different dimensions of dietary quality, the consistent pattern of higher scores across all indices in this group suggests a diet that was not only healthier but also more aligned with sustainability principles in case of the ELD-I.

**Fig. 2:**
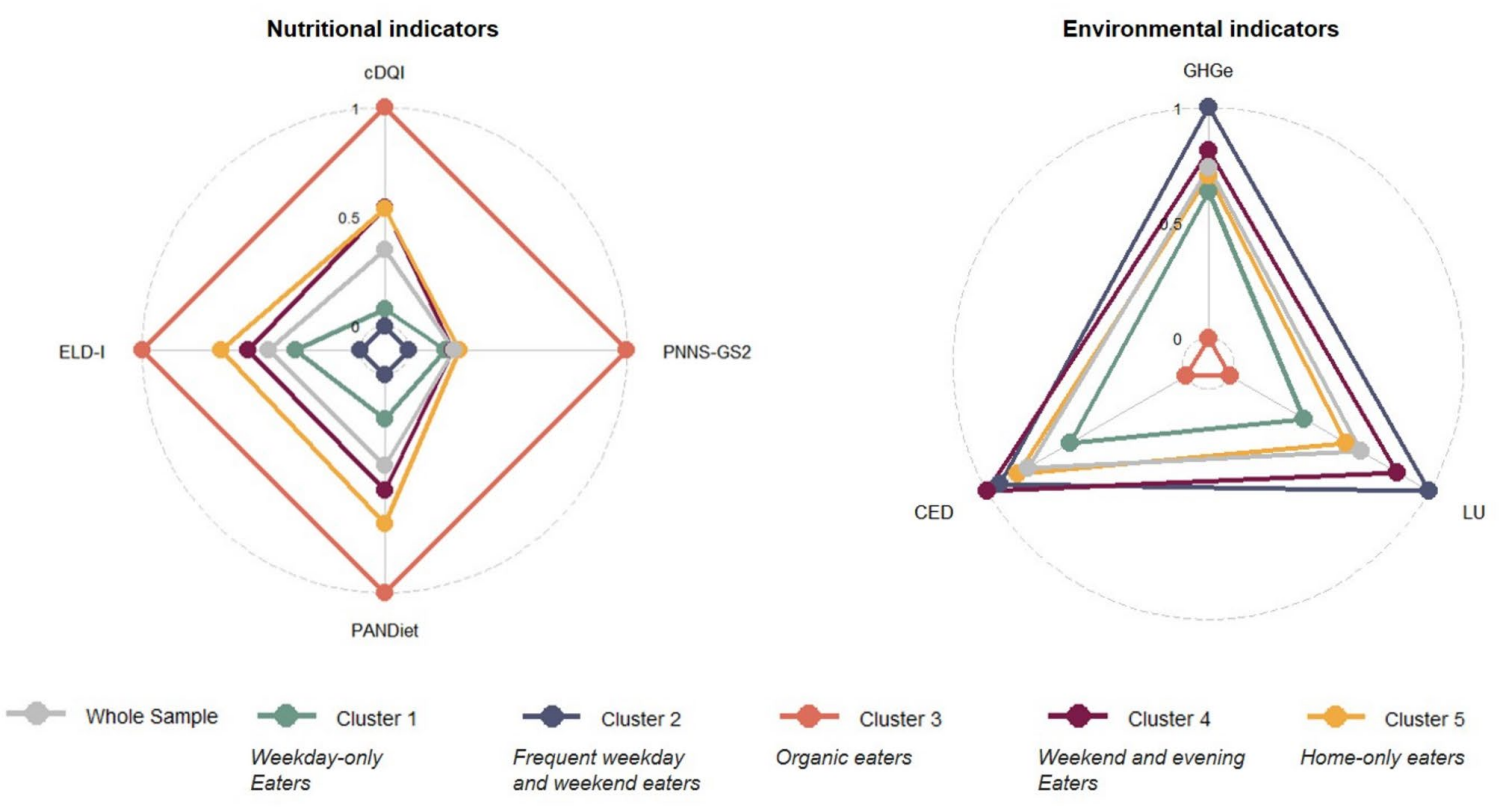
Nutritional and environmental indicators across out-of-home eating behaviour-based clusters. Models were adjusted for energy intake. For each parameter, the adjusted means were rescaled from 0 to 1. For nutritional indicators, higher values represent a higher dietary quality, for environmental indicators higher values indicate higher dietary impacts on the environment. (**GHGe** Greenhouse gas emissions in kg CO_2_-eq/d; **LU** Land use in m^2^/d; **CED** Cumulative energy demand in MJ/d; **cDQI** Comprehensive Diet Quality Index; **PNNS-GS2** Programme National Nutrition Santé-Guidelines Score 2; **PANDiet** Diet Quality Index based on the Probability of Adequate Nutrient Intake; **ELD-I** EAT-Lancet Diet Index)

*Frequent weekday and weekend eaters* showed the lowest scores, indicating a poorer dietary quality (Table 2; Fig. 2). *Organic eaters* showed the lowest meat consumption, with a daily average consumption of ≈ 90 g/d. Also, the lowest dairy product consumption including milk (≈ 210 g/d) and the highest consumption of wholegrain products (≈ 70 g/d) were found in this cluster. In contrast, *Frequent weekday and weekend eaters* showed the lowest intake of fruits and vegetables (≈ 650 g/d) (Supplemental Fig. 2, Additional File). *Organic eaters* further showed the highest amount of organic food within their daily total diet (50.3%) and spent more money on food per day than the other clusters. *Weekday-only eaters*,* Weekend and evening eaters*, and *Home-only eaters* showed intermediate values for all nutritional and cost indicators, i.e., close to the whole sample’s average, with the group of *Home-only eaters* exhibiting a slightly better dietary quality, followed by *Weekend and evening eaters* and then the group of *Weekday-only eaters* (Table 2; Fig. 2).

**Table 2:** Nutritional and dietary cost indicators across out-of-home eating behaviour-based clusters, NutriNet-Santé study (2014, *n* = 29,140)^1,2^

	Cluster 1		Cluster 2		Cluster 3		Cluster 4			Cluster 5		
	Weekday-only eaters		Frequent weekday and weekend eaters		Organic eaters		Weekend and evening eaters			Home-only eaters		
N (%)	*5598 (19.21)*		*6999 (24.02)*		*1820 (6.25)*		*5540 (19.01)*			*9183 (31.51)*		
	Mean	95% CI	Mean	95% CI	Mean	95% CI	Mean	95% CI		Mean	95% CI	P^2^
**PNNS-GS2**												
Model 0^3^	2.55	2.46; 2.65	2.28	2.2; 2.36	3.93	3.78; 4.08	2.41	2.32; 2.5		2.46	2.38; 2.53	< 0.0001
Model 1^4^	2.45	2.37; 2.52	2.16	2.09; 2.23	3.89	3.76; 4.03	2.51	2.43; 2.59		2.56	2.5; 2.62	< 0.0001
Model 2^5^	2.25	2.17; 2.33	1.94	1.86; 2.01	3.6	3.46; 3.73	2.09	2.01; 2.17		2.16	2.1; 2.23	< 0.0001
**PANDiet**												
Model 0^3^	64.67	64.47; 64.88	64.24	64.06; 64.42	66.31	65.95; 66.67	65.07	64.86; 65.28		65.40	65.24; 65.57	< 0.0001
Model 1^4^	64.51	64.32; 64.7	64.06	63.89; 64.23	66.25	65.92; 66.58	65.22	65.03; 65.41		65.56	65.41; 65.71	< 0.0001
Model 2^5^	64.95	64.75; 65.14	64.64	64.46; 64.83	66.58	66.24; 66.91	65.04	64.85; 65.24		65.09	64.92; 65.25	< 0.0001
**ELD-I**												
Model 0^3^	28.54	27.52; 29.56	23.42	22.62; 24.22	40.43	38.66; 42.2	31.93	30.93; 32.93		34.01	33.2; 34.82	< 0.0001
Model 1^4^	28.37	27.38; 29.36	23.23	22.35; 24.11	40.37	38.64; 42.09	32.09	31.1; 33.08		34.18	33.41; 34.95	< 0.0001
Model 2^5^	26.93	25.91; 27.94	21.76	20.82; 22.7	37.67	35.94; 39.4	27.31	26.29; 28.34		29.07	28.24; 29.91	< 0.0001
**cDQI**												
Model 0^3^	50.56	50.32; 50.81	50.30	50.08; 50.52	53.86	53.47; 54.25	52.14	51.9; 52.37		52.12	51.93; 52.3	< 0.0001
Model 1^4^	50.51	50.27; 50.74	50.24	50.03; 50.45	53.84	53.42; 54.26	52.19	51.95; 52.43		52.17	51.98; 52.35	< 0.0001
Model 2^5^	50.19	49.96; 50.42	50.01	49.79; 50.22	52.94	52.54; 53.33	50.09	49.86; 50.33		49.75	49.55; 49.94	< 0.0001
**Share of organic food in diet in %**												
Model 0^3^	29.39	28.69; 30.1	25.08	24.53; 25.63	50.33	49.13; 51.53	29.55	28.82; 30.27		29.07	28.5; 29.65	< 0.0001
Model 1^4^	29.40	28.71; 30.1	25.09	24.47; 25.71	50.33	49.11; 51.56	29.54	28.83; 30.24		29.06	28.52; 29.61	< 0.0001
Model 2^5^	28.39	27.66; 29.11	23.89	23.22; 24.57	48.98	47.74; 50.22	27.99	27.26; 28.72		27.74	27.14; 28.33	< 0.0001
**Dietary monetary cost in €/day**												
Model 0^3^	7.24	7.16; 7.31	7.24	7.17; 7.3	8.29	8.15; 8.43	7.87	7.79; 7.95		7.74	7.68; 7.8	< 0.0001
Model 1^4^	7.35	7.3; 7.4	7.37	7.32; 7.41	8.33	8.24; 8.42	7.76	7.71; 7.81		7.62	7.58; 7.66	< 0.0001
Model 2^5^	7.49	7.44; 7.55	7.57	7.52; 7.62	8.41	8.32; 8.5	7.62	7.56; 7.67		7.36	7.31; 7.4	< 0.0001

**PNNS-GS2** (Programme National Nutrition Santé-Guidelines Score 2): values range from -**∞** and 14.25, a higher score represents better adherence to food-based dietary guidelines

**PANDiet** (Diet Quality Index based on the Probability of Adequate Nutrient Intake): values range from 0 to 100, higher values indicate better nutrient adequacy

**ELD-I** (EAT-Lancet Diet Index): values can be positive or negative, higher values indicate higher adherence to EAT-Lancet diet

**cDQI** (Comprehensive Diet Quality Index): values range from 0 to 85, higher values indicate better dietary quality

^1^ Values are means and 95%-CI

^2^ P-values are based on type III ANCOVA test

^3^ Model 0: unadjusted

^4^ Model 1: adjusted for energy intake

^5^ Model 2: adjusted for energy intake, age and sex

Means of nutritional indicators were not substantially changed after further adjustment for age and sex (model 2) (Table 2).

### Environmental indicators across clusters

In model 1, *Organic eaters* showed the lowest diet-related environmental impacts regarding GHGe (3.6 kg CO_2_-eq/d), CED (16.4 MJ/d), and land use (10.1 m^2^/d). *Frequent weekday and weekend eaters* and *Weekend and evening eaters* had the highest environmental impacts among all clusters while *Weekday-only eaters* and *Home-only eaters* showed intermediate environmental impacts (Table 3; Fig. 2).

**Table 3:** Diet-related environmental indicators across out-of-home eating behaviour-based clusters, NutriNet-Santé study (2014, *n* = 29,140)^1^

	Cluster 1		Cluster 2		Cluster 3			Cluster 4		Cluster 5		
	Weekday-only eaters		Frequent weekday and weekend eaters		Organic eaters			Weekend and evening eaters		Home-only eaters		
N (%)	*5598 (19.21)*		*6999 (24.02)*		*1820 (6.25)*			*5540 (19.01)*		*9183 (31.51)*		
	Mean	95% CI	Mean	95% CI	Mean	95% CI		Mean	95% CI	Mean	95% CI	P^2^
**Greenhouse gas emissions in kg C0**_**2**_ **-eq/d** ^**a**^												
Model 0^3^	3.92	3.85; 3.98	4.12	4.06; 4.18	3.60	3.49; 3.71		4.17	4.1; 4.23	4.11	4.06; 4.16	< 0.0001
Model 1^4^	3.99	3.94; 4.05	4.20	4.16; 4.25	3.63	3.53; 3.72		4.10	4.04; 4.15	4.03	3.99; 4.07	< 0.0001
Model 2^5^	4.16	4.11; 4.22	4.41	4.36; 4.47	3.79	3.7; 3.89		4.17	4.12; 4.23	4.03	3.98; 4.07	< 0.0001
**Cumulative energy demand in MJ/d** ^**b**^												
Model 0^3^	17.01	16.81; 17.21	17.52	17.34; 17.7	16.30	15.96; 16.64		18.22	18.02; 18.41	17.99	17.84; 18.15	< 0.0001
Model 1^4^	17.30	17.17; 17.44	17.85	17.73; 17.97	16.40	16.16; 16.64		17.94	17.81; 18.08	17.70	17.6; 17.81	< 0.0001
Model 2^5^	17.65	17.5; 17.79	18.31	18.18; 18.44	16.67	16.43; 16.91		17.85	17.71; 17.99	17.39	17.27; 17.5	< 0.0001
**Land use in m** ^**2**^ **/d** ^**c**^												
Model 0^3^	10.20	10.02; 10.38	10.63	10.46; 10.79	10.06	9.76; 10.36		10.92	10.75; 11.09	10.75	10.61; 10.88	< 0.0001
Model 1^4^	10.40	10.25; 10.54	10.85	10.72; 10.98	10.13	9.87; 10.39		10.74	10.59; 10.88	10.55	10.44; 10.67	< 0.0001
Model 2^5^	10.86	10.71; 11.01	11.44	11.3; 11.58	10.58	10.32; 10.84		10.90	10.75; 11.05	10.48	10.36; 10.61	< 0.0001

^a^ Greenhouse gas emissions were assessed through life-cycle assessments (from cradle-to-farm). Higher values indicate greater diet-related emissions

^b^ Cumulative energy demand was assessed through life-cycle assessments (from cradle-to-farm). Higher values indicate greater diet-related energy use

^c^ Land use was assessed through life-cycle assessments (from cradle-to-farm). Higher values indicate that greater diet-related land use is required

^1^ Values are means and 95%-CI

^2^ P-values are based on type III ANCOVA test

^3^ Model 0: unadjusted

^4^ Model 1: adjusted for energy intake

^5^ Model 2: adjusted for energy intake, age and sex

Dietary environmental impacts were highest in the group of *Weekend and evening eaters* in the unadjusted model but highest in *Frequent weekday and weekend eaters* cluster after adjustment for energy intake and further adjustment for age and sex (Table 3).

## Discussion

Within our cohort population, five clusters of OOH food consumption were identified based on weekly frequency and type of meals consumed OOH. They were associated with certain sociodemographic, lifestyle, and diet-related characteristics.

### Sociodemographic and lifestyle indicators

*Weekday-only eaters*, *Frequent weekday and weekend eaters*, and *Organic eaters* were characterised by a frequent OOH consumption at lunchtime on weekdays, reflecting the high number of professionally active individuals in these groups, likely eating at their workplace. Previous research has shown that the workplace environment influences nutritional behaviours of individuals [36], which can be supported by our results. *Weekday-only eaters* and *Frequent weekday and weekend eaters* ate more frequently at the canteen than the other groups, indicating a tendency to use workplace offerings. *Organic eaters* tended to opt for restaurants or self-prepared dishes, possibly due to limited availability organic options in canteens.

It has been shown that parents, especially those with younger children, often prioritise family routines, like shared meals, over external social engagements [37]. Our results support these findings, with *Weekday-only eaters* showing the highest proportions of individuals with children at home, likely indicating certain family responsibilities that may limit additional OOH consumption on other occasions during the week. In contrast, *Frequent weekday and weekend eaters* and *Organic eaters*, with smaller proportions of individuals having children at home, showed more frequent OOH consumption on weekends and weekday evenings. In addition, these groups included more singles and individuals with higher socioeconomic status, in line with a previous review examining sociodemographic aspects of OOH consumers across different countries and population groups [5].

*Weekend and evening eaters*, and in particular *Home-only eaters*, comprised the highest numbers of older and retired individuals. *Home-only eaters* showed no OOH consumption and the lowest proportion of high-income individuals, consistent with previous findings from the NutriNet-Santé cohort showing lower overall food intake after retirement [38] or findings from the United States indicating that less money is spent on OOH eating after retirement [39]. *Weekend and evening eaters*, however, showed the second-highest proportion of high-income individuals, likely explaining their higher OOH consumption than *Home-only eaters*, particularly on weekends at restaurants or at family/friends. Since OOH consumption appears to be associated with increased income [5], it can be suggested that this group has the financial means to eat OOH as leisure activity.

While previous findings indicate that frequent OOH eaters are more often male [5], our results revealed higher proportions of women in frequent OOH consumption groups. This discrepancy may result from the sociodemographic composition of our sample, which included an important proportion of women and individuals with a specific interest in nutrition. Another explanation could be the varying definitions of OOH consumption, as in our study, OOH consumption also encompassed self-prepared dishes consumed outside the home, in contrast to previously mentioned research focusing on OOH facilities.

Our findings showed a positive association between frequent OOH consumption and the proportion of urban dwellers (*Frequent weekday and weekend eaters* and *Organic eaters*). This is consistent with previous research linking better access to OOH facilities and a higher frequency of eating OOH in adults across 5 European countries [40]. In line with existing literature, individuals with the highest frequency of organic consumption (*Organic eaters*), were predominantly residents of highly populated urban areas [41, 42]. While the referenced studies focus on specific populations, such as pregnant women in Denmark [41] and a multi-ethnic cohort population in the US [42], both suggest a broader trend of increased organic food consumption in urban environments as they likely provide easier access to organic options. However, the still limited availability of organic and vegetarian options may lead these individuals to rely more often on self-prepared dishes when eating OOH to align their food choices with their dietary motives, ensuring natural and health-conscious options [43, 44]. Furthermore, prior results from the NutriNet-Santé cohort have shown a positive association between vegetarianism and organic food consumption [23, 45]. Our findings are in line with these results, and similar associations have also been confirmed in other populations, such as in the Danish cohort [41].

Previous research links higher socioeconomic status and higher organic consumption to healthier lifestyle, as shown by several studies, including the Danish Diet, Cancer, and Health cohort [46], the NutriNet-Santé cohort [47] and the German National Nutrition Survey [48]. However, in our study, these groups (*Frequent weekday and weekend eaters* and *Organic eaters)* showed the highest proportion of current smokers. In contrast, *Home-only eaters* showed the lowest proportion of current smokers and the highest proportion of former smokers, which aligns with findings from a longitudinal European cohort study across 27 countries indicating a decrease in smoking behaviour after retirement [49].

### Nutritional quality

Our results showed significant differences between clusters with regard to nutritional quality. Most frequent OOH eaters (*Frequent weekday and weekend eaters*) had a lower overall nutritional quality, in line with recent reviews that showed lower nutritional quality among regular OOH consumers [5, 8]. Frequent OOH consumption is associated with higher energy and total fat intake, and higher BMI but with lower micronutrient intake, as shown by different reviews [5, 7, 8] and the EPIC cohort study across 10 western European countries [50]. Furthermore, findings suggest that meals considered as healthy are generally consumed at home rather than away from home [5]. Similarly, *Weekday-only eaters*, with a high frequency of eating OOH at lunchtime on weekdays, showed the second lowest dietary quality while clusters with lower OOH frequency showed higher nutritional quality. In the present study, we observed that *Organic eaters* had the highest dietary quality, despite relatively high OOH consumption. It has been shown that organic eaters generally have healthier dietary habits [23, 41, 45, 47, 48]. In our study, the group of *Organic eaters* showed the highest consumption of organic products, further supporting the hypothesis that this group is particularly health-conscious.

### Environmental indicators

A high frequency of eating OOH was related to higher diet-related environmental impacts. Thus, *Frequent weekday and weekend eaters* had the highest diet-related GHGe and LU. This can be explained by their high intake of meat and dairy products in combination with a low fruit and vegetable consumption. The high climate and land footprints of animal-based foods, in particular ruminant meat and dairy products, are now well documented [51, 52]. However, *Weekend and evening eaters* showed the highest CED and thus exhibited higher environmental impacts but better dietary quality than the whole sample’s average. A possible explanation could be that the adherence of dietary recommendations is not always associated with lower environmental impacts as shown by previous research comparing the health and environmental impacts of different food-based dietary guidelines [53]. Unsurprisingly, individuals with the highest consumption of plant-based products and the lowest consumption of meat-based products (*Organic eaters*) showed the lowest diet-related environmental impacts, in line with scientific evidence [54–56].

### Levers for the transition towards more sustainable and healthier diets

OOH consumption has gained importance in recent years, with 80% of French adults eating OOH at least once a week in 2014 [4], while 72% of our study sample did so. Therefore, the OOH sector can be seen as a promising lever towards healthier and more sustainable dietary patterns since individual food choices depend on availability and offers.

Recent reviews highlight that most effective changes towards healthier and more environmentally friendly diets include interventions such as consumers’ education or reorganising settings [57]. For instance, these approaches could include offering healthier and more sustainable meals in OOH facilities. In this context, public catering plays a crucial role in promoting sustainable and healthy diets by offering large-scale intervention possibilities across various settings and population groups, at reasonable monetary cost. Since meals provided in public catering, such as those in canteens, are often “default choices” for consumers, the menu design seems to be important for integrating sustainable and healthy food into everyday life. Initiatives such as the “Lundi vert” (Green Monday) [58] or previous experimental studies in public catering settings [59, 60], offering more plant-based foods or nutrient information, have shown positive impacts on more sustainable, healthy diets. Providing healthy and sustainable food choices in the OOH sector, especially in public catering, could help to reduce inequalities in access to healthy and sustainable food. In this context, initiatives, such as the previously mentioned French regulation EGalim that aims for 50% sustainable food and at least one vegetarian lunch per week [61], seem relevant.

Moreover, promoting sustainable OOH food consumption is crucial, as meals served in these settings may influence home eating habits by offering healthy and sustainable meal ideas. However, intervention targets could differ between individuals who prepare their meals at home but consume it outside the home and those who eat and buy OOH.

### Strengths and limitations

A key strength of this study is its large and diverse sample, enabling detailed identification of different OOH consumption clusters and their associated sociodemographic, nutritional, and environmental indicators. However, data from self-reported questionnaires could lead to bias. In addition, participation in the cohort was on a voluntary basis, so participants may have more interest in nutrition and health-related topics than the general population [62]. Despite the large and diverse sample, the typology cannot be generalised to the general French population as the study population included more females, older individuals, and those with higher educational. Dietary environmental impacts of the diets were limited to food production stage, where the majority of environmental impacts occur [63], except for CED. Furthermore, the study relied on 2014 data, as complete sustainability data was only available for this year. Another limitation is that breakfast as an OOH eating occasion was not assessed within the questionnaire used. Finally, diets were assessed using an FFQ, so the investigated indicators were based on participants’ entire diets (including OOH consumption). It was not possible to distinguish which foods were consumed OOH or at home. Additionally, the questionnaire about eating practices, attitudes, and motivations was not formally validated for this study, but it has been used in previous studies [15, 16].

Nevertheless, our study provides valuable insights by considering both environmental and nutritional aspects, allowing a more holistic understanding of OOH consumers.

## Conclusions

We identified five OOH consumption clusters based on their eating patterns at different occasions of the week. Frequent OOH consumers tended to be younger with higher socioeconomic status, but showed a poorer nutritional quality and higher diet-related environmental impacts. In contrast, individuals showing the highest consumption of organic food when eating out, showed the best nutritional quality and lowest environmental impacts.

As OOH consumption varies across population groups, public health strategies should target both consumers and food offerings, adapting to distinct sociodemographic profiles. Moreover, further longitudinal studies are needed to assess the impact of OOH consumption considering the type of settings (e.g. fast food restaurants, canteens) on both long-term health and the environment.

## Electronic supplementary material

Below is the link to the electronic supplementary material.

Supplementary Material 1: **Additional File** contains **Supplemental Method 1**: Computation of dietary scores; **Supplemental Fig. 1**: Study sample selection; **Supplemental Method 2**: Construction of OOH consumption typology; **Supplemental Table 1**: Values of OOH typology constructing variables; **Supplemental Fig. 2**: Food group consumption across clusters.

## Data Availability

Analytic code will be made available upon request pending. Researchers from public institutions can submit a collaboration request including information on the institution and a brief description of the project to collaboration@etude-nutrinet-sante.fr. All requests will be reviewed by the steering committee of the NutriNet-Santé study. If the collaboration is accepted, a data access agreement will be necessary and appropriate authorizations from the competent administrative authorities may be needed. In accordance with existing regulations, no personal data will be accessible.

## Abbreviations

BMI: Body mass index
CED: Cumulative energy demand
cDQI: Comprehensive Diet Quality Index
ELD-I: EAT-Lancet Diet Index
GHGe: Greenhouse gas emissions
LU: Land use
MFA: Multiple Factor Analysis
Org-FFQ: Organic food frequency questionnaire
OOH: Out-of-home
PANDiet: Diet Quality Index based on the Probability of Adequate Nutrient Intake
PNNS-GS2: Programme National Nutrition Santé-Guidelines Score 2
