# Supplementary material for: Typology of out-of-home eaters: a description of sociodemographic, lifestyle, nutritional and environmental characteristics in the NutriNet-Santé cohort
Source: Int J Behav Nutr Phys Act. 2025 May 26;22:61. doi: 10.1186/s12966-025-01752-5 (PMC12107787; doi:10.1186/s12966-025-01752-5)
Supplement: Supplementary file 1 — Supplementary Material 1: Additional File contains Supplemental Method 1: Computation of dietary scores; Supplemental Fig. 1: Study sample selection; Supplemental Method 2: Construction of OOH consumption typology; Supplemental Table 1: Values of OOH typology constructing variables; Supplemental Fig. 2: Food group consumption across clusters. [file 12966_2025_1752_MOESM1_ESM.docx]

# Additional File

# **[Supplemental Method 1](#_Supplemental_Method_1:_1)**[: Computation of dietary scores](#_Supplemental_Method_1:_1)

# [**Supplemental Figure 1**: Study sample selection](#_Supplemental_Figure_1)

# **[Supplemental Method 2](#_Supplemental_Method_2:_1)**[: Construction of OHH consumption typology](#_Supplemental_Method_2:_1)

# **[Supplemental Table 1](#_Supplemental_Figure_1:_1)**[: Values of OOH-typology constructing variables](#_Supplemental_Figure_1:_1)

# **[Supplemental Figure 2:](#_Supplemental_Figure_2)** [Food group consumption across clusters](#_Supplemental_Figure_2)

# **Supplemental Method 1**: Computation of dietary scores

The ***PNNS-GS2*** (National Nutrition Health Guidelines Score) is a score aiming to measure the adherence of individuals’ diets to the official French dietary recommendations. For calculation, 13 dietary components are considered. 7 are considered beneficial (fruits and vegetables, nuts, legumes, whole grains, milk and dairy products, fish and seafood, and added fats), and 6 are deemed unfavourable (red meat, processed meat, sweets, sweetened beverages, alcoholic beverages, and salt). For each positive component, scores ranging from 0 to 2 are assigned, while negative components are scored from 0 to -2, based on average French portion sizes. Additionally, the consumption of organic foods is considered for certain categories (fruits, vegetables, legumes, and whole-grain foods). Adherence to the recommendations is assessed by assigning scores to the intake of each food group. Weighting factors are applied to food groups based on their health impact. The score is penalised on energy intake if an energy intake of more than 105% of energy expenditure is detected, which means that the score is reduced by the same proportion. The total score can range from -∞ to 14.25, with higher scores indicating better dietary quality (1).

The ***PANDiet*** (Diet Quality Index based on the Probability of Adequate Nutrient Intake) score measures nutrient adequacy. A total of 28 nutrients are used for calculation, building two sub-scores, the Adequacy Sub-score (AS) and the Moderation Sub-score (MS). The AS presents the adequacy for 28 nutrients (protein, total fat, Linolenic acid, α-Linolenic acid, Eicosapentaenoic acid, Docosahexaenoic acid, fibre, vitamins (A, B1, B2, B3, B5, B6, B9, B12, C, D and E), calcium, copper, iodine, bioavailable iron, magnesium, manganese, bioavailable zinc, phosphorus, potassium, selenium) whose intake should meet a certain reference while the MS concerns the average adherence probability for 6 nutrients (protein, total fat, sugars, saturated fatty acids, cholesterol, and sodium) that should not exceed a certain value. The latter includes 12 penalty values if the limits are exceeded. Computed values range between 0 and 100, higher value indicates better dietary quality (2).

The ***cDQI*** (Comprehensive Diet Quality Index) comprises the plant-based diet quality index (pDQI) and the animal-based diet quality index (aDQI). The computation is based on 17 food groups (11 plant-based foods and 6 animal-based foods). Different food groups are considered depending on their healthful or unhealthy contribution. Healthful plant-based food groups include wholegrain products, vegetables (excluding potatoes), fruits, nuts, seeds, legumes, vegetable oils, coffee and tea, while unhealthy components include fruit juices, refined grains, potatoes, sugar-sweetened beverages, sweets and desserts. Fish, seafood, dairy and poultry are considered animal-based healthful food groups. Processed meat, red meat and eggs are counted as unhealthy animal-based components. Depending on their consumption quantity, scores from 0 to 5 per food group are assigned. The score increases (for favourable components) and decreases (for unfavourable components) proportionally using quintiles from no consumption to maximum consumption. Thus, the calculated score ranges from 0 to 85, a higher score indicates higher dietary quality of both animal-based and plant-based components (3).

The ***ELD-I*** (EAT-Lancet Diet Index) represents the dietary adherence to the EAT-Lancet diet. This diet promotes increased quantities of plant-based foods in diets with positive impacts on human and planet health (4). It comprises the following 14 food groups: potatoes and tubers, fruits, vegetables, whole grains, dairy, beef/lamb/pork, chicken and other poultry, eggs, fish, legumes, nuts, saturated oil, unsaturated oils, and added sugar. The score is calculated based on consumed quantities of the respective food groups and is displayed as continuous values that can be negative or positive. The higher the values, the higher the adherence to the EAT-Lancet diet (5).

# **Supplemental Figure 1**: Study sample selection

N = 40,680

N = 29,140

Had no missing data on OOH variables

**Questionnaire on eating practices, attitudes and motivations**

**Organic Food-Frequency-Questionnaire**

Final sample

N = 37,685

Completed the questionnaire

N = 37,307

Had no missing covariates

N = 35,196

Were not under/over reporters

N = 34,453

Were not living overseas

N = 29,210

Had available data for investigated indicators

# **Supplemental Method 2**: Construction of OHH consumption typology

**Scree plot as decision criterion for number of dimensions to retain in MFA:**

**
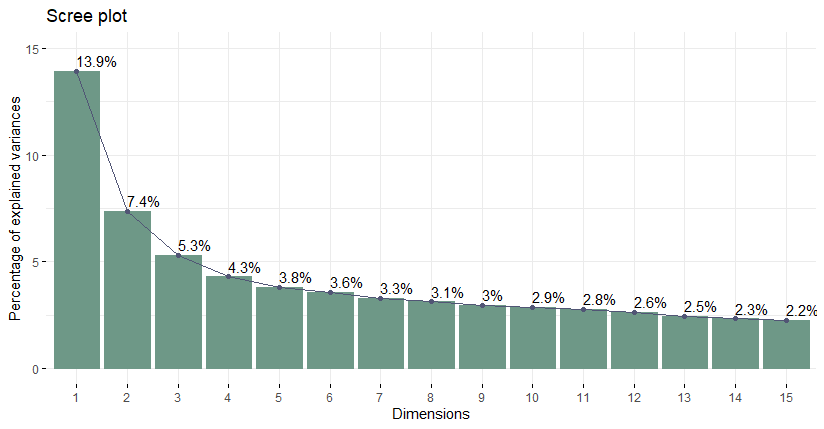
**

**Factor Loadings of quantitative variables**

| **Description** | **Variable** | **Dim.1** | **Dim.2** | **Dim.3** |
| --- | --- | --- | --- | --- |
| Number of times per week eating OOH at lunchtime on weekdays | num_hd_midi_sem | 1.0979349 | -1.3495018 | -0.6989206 |
| Number of times per week eating OOH in the evening on weekdays | num_hd_soir_sem | 0.3819118 | 0.22706 | -0.1851816 |
| Number of times per week eating OOH on weekends | num_hd_we | 0.4601147 | 0.2833144 | -0.2369407 |

**Factor Loadings of qualitative variables (per category)**

| **Description of each variable** | | | **Variable** | **Dim.1** | **Dim.2** | **Dim.3** |
| --- | --- | --- | --- | --- | --- | --- |
| Frequently | Self-prepared meals | At lunchtime on weekdays | mi_sem_prep_fré | 0.91652301 | -0.80416848 | -0.420736219 |
| Never | Self-prepared meals | At lunchtime on weekdays | mi_sem_prep_jam | -0.65400096 | 0.4032676 | 0.059511818 |
| Sometimes | Self-prepared meals | At lunchtime on weekdays | mi_sem_prep_par | 1.53020523 | -0.68624941 | 0.223883058 |
| Frequently | Restaurant meals | At lunchtime on weekdays | mi_sem_rest_fré | 0.77427171 | -0.0401285 | 0.030920988 |
| Never | Restaurant meals | At lunchtime on weekdays | mi_sem_rest_jam | -0.76110683 | 0.34296612 | 0.021088852 |
| Sometimes | Restaurant meals | At lunchtime on weekdays | mi_sem_rest_par | 1.42700458 | -0.80203884 | -0.066511333 |
| Frequently | Take-away meals | At lunchtime on weekdays | mi_sem_emp_fré | 1.03793615 | -0.65017111 | -0.206092341 |
| Never | Take-away meals | At lunchtime on weekdays | mi_sem_emp_jam | -0.59653487 | 0.32104624 | 0.028114158 |
| Sometimes | Take-away meals | At lunchtime on weekdays | mi_sem_emp_par | 1.48145726 | -0.7802272 | -0.040517713 |
| Frequently | Delivery meals | At lunchtime on weekdays | mi_sem_liv_fré | 1.88826522 | -0.988284 | 0.927676608 |
| Never | Delivery meals | At lunchtime on weekdays | mi_sem_liv_jam | -0.07186174 | 0.02583433 | -0.035266059 |
| Sometimes | Delivery meals | At lunchtime on weekdays | mi_sem_liv_par | 2.53705136 | -0.88141581 | 1.244954376 |
| Frequently | Canteen meals | At lunchtime on weekdays | mi_sem_can_fré | 0.74987836 | -0.72995996 | -0.71848131 |
| Never | Canteen meals | At lunchtime on weekdays | mi_sem_can_jam | -0.34913919 | 0.25106871 | 0.129048182 |
| Sometimes | Canteen meals | At lunchtime on weekdays | mi_sem_can_par | 1.53800554 | -0.82471763 | 0.082385324 |
| Frequently | Meals at family/friends | At lunchtime on weekdays | mi_sem_fam_fré | 1.07404951 | 0.2859967 | -0.085712216 |
| Never | Meals at family/friends | At lunchtime on weekdays | mi_sem_fam_jam | -0.40405359 | 0.0714327 | -0.026993118 |
| Sometimes | Meals at family/friends | At lunchtime on weekdays | mi_sem_fam_par | 1.39152435 | -0.36366709 | 0.131894567 |
| Frequently | Self-prepared meals | At evenings on weekdays or weekends | soir_we_prep_fré | 1.18601351 | 0.83106801 | -0.554457804 |
| Never | Self-prepared meals | At evenings on weekdays or weekends | soir_we_prep_jam | -0.73200843 | -0.38758246 | 0.126569841 |
| Sometimes | Self-prepared meals | At evenings on weekdays or weekends | soir_we_prep_par | 1.57193509 | 0.63949597 | 0.059884998 |
| Frequently | Restaurant meals | At evenings on weekdays or weekends | soir_we_rest_fré | 0.94407139 | 0.6612226 | -0.332922961 |
| Never | Restaurant meals | At evenings on weekdays or weekends | soir_we_rest_jam | -1.0660458 | -0.47472139 | 0.241735755 |
| Sometimes | Restaurant meals | At evenings on weekdays or weekends | soir_we_rest_par | 1.39876372 | 0.42534414 | -0.219694563 |
| Frequently | Take-away meals | At evenings on weekdays or weekends | soir_we_emp_fré | 1.17741524 | 0.8458355 | -0.699450171 |
| Never | Take-away meals | At evenings on weekdays or weekends | soir_we_emp_jam | -0.39660721 | -0.17935077 | 0.0325419 |
| Sometimes | Take-away meals | At evenings on weekdays or weekends | soir_we_emp_par | 1.69092433 | 0.73212147 | -0.076155824 |
| Frequently | Delivery meals | At evenings on weekdays or weekends | soir_we_liv_fré | 1.65354593 | 0.67653946 | -0.6926557 |
| Never | Delivery meals | At evenings on weekdays or weekends | soir_we_liv_jam | -0.14559055 | -0.03980267 | -0.00568255 |
| Sometimes | Delivery meals | At evenings on weekdays or weekends | soir_we_liv_par | 2.11745506 | 0.55722473 | 0.155712983 |
| Frequently | Canteen meals | At evenings on weekdays or weekends | soir_we_can_fré | 1.13231129 | 0.9971186 | -1.215189476 |
| Never | Canteen meals | At evenings on weekdays or weekends | soir_we_can_jam | -0.09699374 | -0.06591158 | 0.000788183 |
| Sometimes | Canteen meals | At evenings on weekdays or weekends | soir_we_can_par | 1.78591467 | 1.15308863 | 0.306078519 |
| Frequently | Meals at family/friends | At evenings on weekdays or weekends | soir_we_fam_fré | 1.12353957 | 0.62687676 | -0.427255304 |
| Never | Meals at family/friends | At evenings on weekdays or weekends | soir_we_fam_jam | -1.10034617 | -0.50111231 | 0.266687427 |
| Sometimes | Meals at family/friends | At evenings on weekdays or weekends | soir_we_fam_par | 1.25352342 | 0.44707911 | -0.137312011 |
| Frequently | Self-prepared meals organic | At lunchtime on weekdays | midi_sem_prep_bio_fré | 1.33066054 | -0.90647592 | 0.45467207 |
| Never | Self-prepared meals organic | At lunchtime on weekdays | midi_sem_prep_bio_jam | -0.44888183 | 0.24353317 | -0.097139953 |
| Sometimes | Self-prepared meals organic | At lunchtime on weekdays | midi_sem_prep_bio_par | 1.70389992 | -0.84141858 | 0.293750631 |
| Frequently | Restaurant meals organic | At lunchtime on weekdays | midi_sem_rest_bio_fré | 1.80079375 | -0.27663873 | 1.625758565 |
| Never | Restaurant meals organic | At lunchtime on weekdays | midi_sem_rest_bio_jam | -0.22572971 | 0.05857231 | -0.15503679 |
| Sometimes | Restaurant meals organic | At lunchtime on weekdays | midi_sem_rest_bio_par | 2.36580056 | -0.62429415 | 1.603638727 |
| Frequently | Take-away meals organic | At lunchtime on weekdays | midi_sem_emp_bio_fré | 1.36007416 | -1.07533557 | 0.97232525 |
| Never | Take-away meals organic | At lunchtime on weekdays | midi_sem_emp_bio_jam | -0.22489377 | 0.08432246 | -0.138950433 |
| Sometimes | Take-away meals organic | At lunchtime on weekdays | midi_sem_emp_bio_par | 2.51320735 | -0.90094115 | 1.543124218 |
| Frequently | Delivery meals organic | At lunchtime on weekdays | midi_sem_livr_bio_fré | 2.38036752 | -2.11709951 | 3.065471865 |
| Never | Delivery meals organic | At lunchtime on weekdays | midi_sem_livr_bio_jam | -0.0314375 | 0.01018592 | -0.039381842 |
| Sometimes | Delivery meals organic | At lunchtime on weekdays | midi_sem_livr_bio_par | 4.16682572 | -1.24704304 | 5.213395387 |
| Frequently | Self-prepared meals organic | At evenings on weekdays or weekends | soir_we_prep_bio_fré | 1.66257925 | 1.0566712 | 0.318138717 |
| Never | Self-prepared meals organic | At evenings on weekdays or weekends | soir_we_prep_bio_jam | -0.4388197 | -0.22933019 | -0.059404619 |
| Sometimes | Self-prepared meals organic | At evenings on weekdays or weekends | soir_we_prep_bio_par | 1.87480542 | 0.94893597 | 0.238510237 |
| Frequently | Restaurant meals organic | At evenings on weekdays or weekends | soir_we_rest_bio_fré | 2.04816298 | 0.81997477 | 1.020974006 |
| Never | Restaurant meals organic | At evenings on weekdays or weekends | soir_we_rest_bio_jam | -0.27631441 | -0.10344825 | -0.135697952 |
| Sometimes | Restaurant meals organic | At evenings on weekdays or weekends | soir_we_rest_bio_par | 2.33312255 | 0.8704076 | 1.144919869 |
| Frequently | Take-away meals organic | At evenings on weekdays or weekends | soir_we_emp_bio_fré | 1.67972556 | 1.50237679 | 0.223825216 |
| Never | Take-away meals organic | At evenings on weekdays or weekends | soir_we_emp_bio_jam | -0.15200213 | -0.07254448 | -0.091750018 |
| Sometimes | Take-away meals organic | At evenings on weekdays or weekends | soir_we_emp_bio_par | 2.83900806 | 1.33226256 | 1.73922794 |
| Frequently | Delivery meals organic | At evenings on weekdays or weekends | soir_we_livr_bio_fré | 1.43982394 | 2.30714573 | -0.286908282 |
| Never | Delivery meals organic | At evenings on weekdays or weekends | soir_we_livr_bio_jam | -0.03560199 | -0.01377432 | -0.036120654 |
| Sometimes | Delivery meals organic | At evenings on weekdays or weekends | soir_we_livr_bio_par | 4.42980201 | 1.66061622 | 4.547527675 |

**Scree plot as decision criterion for the optimal number of clusters:**

**
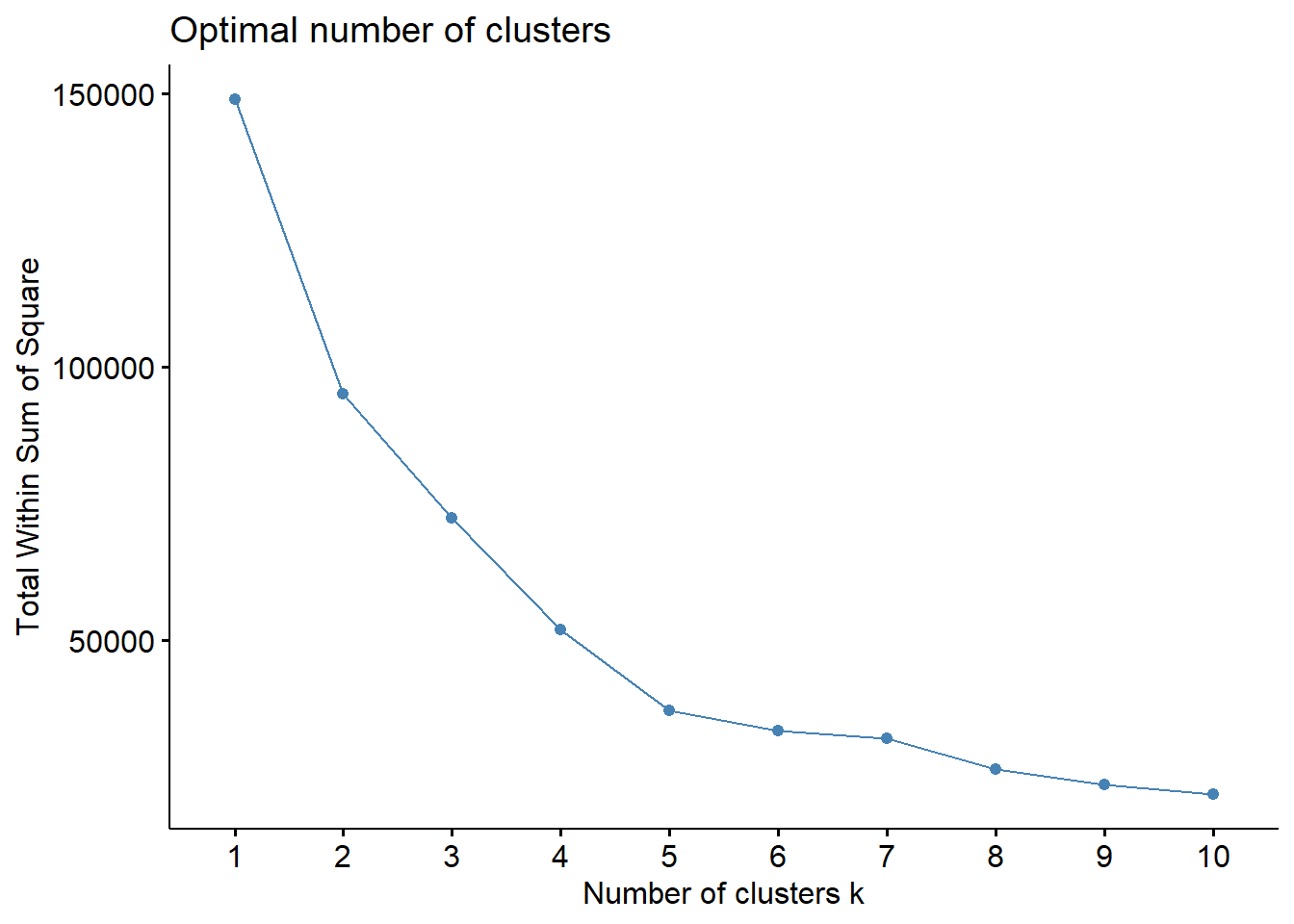
**

# **Supplemental Table 1**: Values of OOH-typology constructing variables NutriNet-Santé study (2014, n = 29,140)^1^

|  |  | **Cluster 1** | | **Cluster 2** | | **Cluster 3** | | **Cluster 4** | | **Cluster 5** | |
| --- | --- | --- | --- | --- | --- | --- | --- | --- | --- | --- | --- |
|  |  | **Weekday-only eaters** | | **Frequent weekday and weekend eaters** | | **Organic eaters** | | **Weekend and evening eaters** | | **Home-only eaters** | |
| N | | *5598* | | *6999* | | *1820* | | *5540* | | *9183* | |
| % | | *19.21* | | *24.02* | | *6.25* | | *19.01* | | *31.51* | |
|  |  | Mean | 95% CI | Mean | 95% CI | Mean | 95% CI | Mean | 95% CI | Mean | 95% CI |
| **Absolute number of times of eating OOH (times per week)** | | | | | | | | | | | |
| At lunchtime on weekdays | | 3.81 | 3.77 ; 3.85 | 3.63 | 3.59 ; 3.66 | 2.84 | 2.76 ; 2.92 | 0.3 | 0.29 ; 0.32 | 0.13 | 0.12 ; 0.14 |
| In the evening on weekdays | | 0.02 | 0.02 ; 0.02 | 0.83 | 0.8 ; 0.85 | 0.89 | 0.85 ; 0.92 | 0.63 | 0.61 ; 0.65 | 0.01 | 0.01 ; 0.01 |
| On weekends | | 0.05 | 0.04 ; 0.05 | 1.16 | 1.15 ; 1.17 | 1.1 | 1.07 ; 1.12 | 1.03 | 1.02 ; 1.05 | 0.02 | 0.01 ; 0.02 |
|  |  |  |  |  |  |  |  |  |  |  |  |
|  | | **Cluster 1** | | **Cluster 2** | | **Cluster 3** | | **Cluster 4** | | **Cluster 5** | |
| N | | *5598* | | *6999* | | *1820* | | *5540* | | *9183* | |
| % | | *19.21* | | *24.02* | | *6.25* | | *19.01* | | *31.51* | |
| **Type of meal at lunchtime on weekdays when eating OOH** | | | | | | | | | | | |
| *Summarised frequency in %* | | | | | | | | | | | |
| Self-prepared | |  | |  | |  | |  | |  | |
|  | Frequently | 36.42 | | 36.29 | | 22.36 | | 3.01 | | 1.55 | |
|  | Sometimes | 27.63 | | 31.72 | | 52.47 | | 2.56 | | 0.59 | |
|  | Never | 35.94 | | 31.99 | | 25.16 | | 94.42 | | 97.87 | |
| Canteen | |  | |  | |  | |  | |  | |
|  | Frequently | 28.87 | | 31.23 | | 13.19 | | 2.38 | | 1.12 | |
|  | Sometimes | 17.17 | | 19.49 | | 27.25 | | 0.97 | | 0.27 | |
|  | Never | 53.97 | | 49.28 | | 59.56 | | 96.64 | | 98.61 | |
| Restaurant | |  | |  | |  | |  | |  | |
|  | Frequently | 14.13 | | 17.42 | | 26.43 | | 15.00 | | 5.58 | |
|  | Sometimes | 41.25 | | 52.49 | | 63.35 | | 3.86 | | 1.05 | |
|  | Never | 44.62 | | 30.09 | | 10.22 | | 81.14 | | 93.38 | |
| Delivery | |  | |  | |  | |  | |  | |
|  | Frequently | 0.41 | | 0.36 | | 1.26 | | 0.04 | | 0.01 | |
|  | Sometimes | 2.59 | | 4.66 | | 14.40 | | 0.20 | | 0.03 | |
|  | Never | 97.00 | | 94.99 | | 84.34 | | 99.77 | | 99.96 | |
| Take away | |  | |  | |  | |  | |  | |
|  | Frequently | 8.13 | | 8.52 | | 8.57 | | 1.59 | | 0.71 | |
|  | Sometimes | 40.46 | | 51.36 | | 65.22 | | 3.16 | | 0.81 | |
|  | Never | 51.41 | | 40.12 | | 26.21 | | 95.25 | | 98.49 | |
| At family / friends | | | |  | |  | |  | |  | |
|  | Frequently | 3.70 | | 7.20 | | 10.82 | | 5.83 | | 1.27 | |
|  | Sometimes | 22.49 | | 35.46 | | 51.92 | | 9.01 | | 2.86 | |
|  | Never | 73.81 | | 57.34 | | 37.25 | | 85.16 | | 95.86 | |
| **Type of meal in the evening on weekdays or on weekends when eating OOH** | | | | | | | | | | | |
| *Summarised frequency in %* | | | | | | | | | | | |
| Self-prepared | |  | |  | |  | |  | |  | |
|  | Frequently | 0.61 | | 33.25 | | 24.34 | | 37.49 | | 0.32 | |
|  | Sometimes | 0.46 | | 33.69 | | 53.08 | | 32.56 | | 0.20 | |
|  | Never | 98.93 | | 33.06 | | 22.58 | | 29.95 | | 99.49 | |
| Canteen | |  | |  | |  | |  | |  | |
|  | Frequently | 0.05 | | 2.70 | | 0.88 | | 2.36 | | 0.01 | |
|  | Sometimes | 0.09 | | 6.84 | | 14.34 | | 9.55 | | 0.05 | |
|  | Never | 99.86 | | 90.46 | | 84.78 | | 88.09 | | 99.93 | |
| Restaurant | |  | |  | |  | |  | |  | |
|  | Frequently | 3.20 | | 35.31 | | 34.34 | | 49.95 | | 1.66 | |
|  | Sometimes | 2.18 | | 56.34 | | 59.51 | | 42.33 | | 0.65 | |
|  | Never | 94.62 | | 8.36 | | 6.15 | | 7.73 | | 97.69 | |
| Delivery | |  | |  | |  | |  | |  | |
|  | Frequently | 0.02 | | 1.40 | | 1.04 | | 0.90 | | 0.00 | |
|  | Sometimes | 0.14 | | 13.10 | | 22.31 | | 7.38 | | 0.01 | |
|  | Never | 99.84 | | 85.50 | | 76.65 | | 91.71 | | 99.99 | |
| Take away | |  | |  | |  | |  | |  | |
|  | Frequently | 0.20 | | 3.60 | | 2.69 | | 3.92 | | 0.04 | |
|  | Sometimes | 0.32 | | 34.38 | | 49.89 | | 32.31 | | 0.14 | |
|  | Never | 99.48 | | 62.02 | | 47.42 | | 63.77 | | 99.81 | |
| At family / friends | | | | | | | | | | | |
|  | Frequently | 2.34 | | 47.66 | | 41.92 | | 53.45 | | 0.79 | |
|  | Sometimes | 2.48 | | 46.95 | | 51.37 | | 41.61 | | 1.03 | |
|  | Never | 95.18 | | 5.39 | | 6.70 | | 4.95 | | 98.17 | |
|  | | | | | | | | | | | |
| **Type of meal at lunchtime on weekdays when eating OOH (organic)** | | | | | | | | | | | |
| *Summarised frequency in %* | | | | | | | | | | | |
| Self-prepared | |  | |  | |  | |  | |  | |
|  | Frequently | 14.33 | | 9.64 | | 23.19 | | 0.83 | | 0.51 | |
|  | Sometimes | 27.37 | | 27.39 | | 52.64 | | 0.34 | | 0.04 | |
|  | Never | 58.31 | | 62.97 | | 24.18 | | 98.83 | | 99.44 | |
| Restaurant | |  | |  | |  | |  | |  | |
|  | Frequently | 0.54 | | 0.19 | | 3.30 | | 0.27 | | 0.16 | |
|  | Sometimes | 11.65 | | 4.90 | | 70.16 | | 2.08 | | 0.52 | |
|  | Never | 87.82 | | 94.91 | | 26.54 | | 97.65 | | 99.31 | |
| Delivery | |  | |  | |  | |  | |  | |
|  | Frequently | 0.16 | | 0.00 | | 0.38 | | 0.00 | | 0.00 | |
|  | Sometimes | 0.16 | | 0.00 | | 10.93 | | 0.00 | | 0.01 | |
|  | Never | 99.68 | | 100.00 | | 88.68 | | 100.00 | | 99.99 | |
| Take away | |  | |  | |  | |  | |  | |
|  | Frequently | 1.39 | | 0.36 | | 2.69 | | 0.20 | | 0.07 | |
|  | Sometimes | 12.43 | | 6.37 | | 62.14 | | 0.31 | | 0.08 | |
|  | Never | 86.17 | | 93.27 | | 35.16 | | 99.49 | | 99.86 | |
| **Type of meal in the evening on weekdays or on week-ends when eating OOH (organic)** | | | | | | | | | | | |
| *Summarised frequency in %* | | | | | | | | | | | |
| Self-prepared | |  | |  | |  | |  | |  | |
|  | Frequently | 0.09 | | 3.83 | | 10.05 | | 5.97 | | 0.03 | |
|  | Sometimes | 0.07 | | 26.09 | | 63.90 | | 32.80 | | 0.01 | |
|  | Never | 99.84 | | 70.08 | | 26.04 | | 61.23 | | 99.96 | |
| Restaurant | |  | |  | |  | |  | |  | |
|  | Frequently | 0.04 | | 0.51 | | 3.85 | | 1.08 | | 0.02 | |
|  | Sometimes | 0.32 | | 8.66 | | 71.98 | | 17.96 | | 0.05 | |
|  | Never | 99.64 | | 90.83 | | 24.18 | | 80.96 | | 99.92 | |
| Delivery | |  | |  | |  | |  | |  | |
|  | Frequently | 0.00 | | 0.01 | | 0.05 | | 0.09 | | 0.00 | |
|  | Sometimes | 0.00 | | 0.07 | | 11.59 | | 0.25 | | 0.00 | |
|  | Never | 100.00 | | 99.91 | | 88.35 | | 99.66 | | 100.00 | |
| Take away | |  | |  | |  | |  | |  | |
|  | Frequently | 0.02 | | 0.13 | | 0.71 | | 0.43 | | 0.00 | |
|  | Sometimes | 0.00 | | 2.44 | | 43.02 | | 9.01 | | 0.00 | |
|  | Never | 99.98 | | 97.43 | | 56.26 | | 90.56 | | 100.00 | |
| ^1^ Values are means (SD) for continuous variables and percentages for categorical variables.  P-values are based on type III ANOVA test or χ2 test, as appropriate, all P-values < 0.0001  Data is unadjusted. | | | | | | | | | | | |

# **Supplemental Figure 2:** Food group consumption across clusters (g/d)


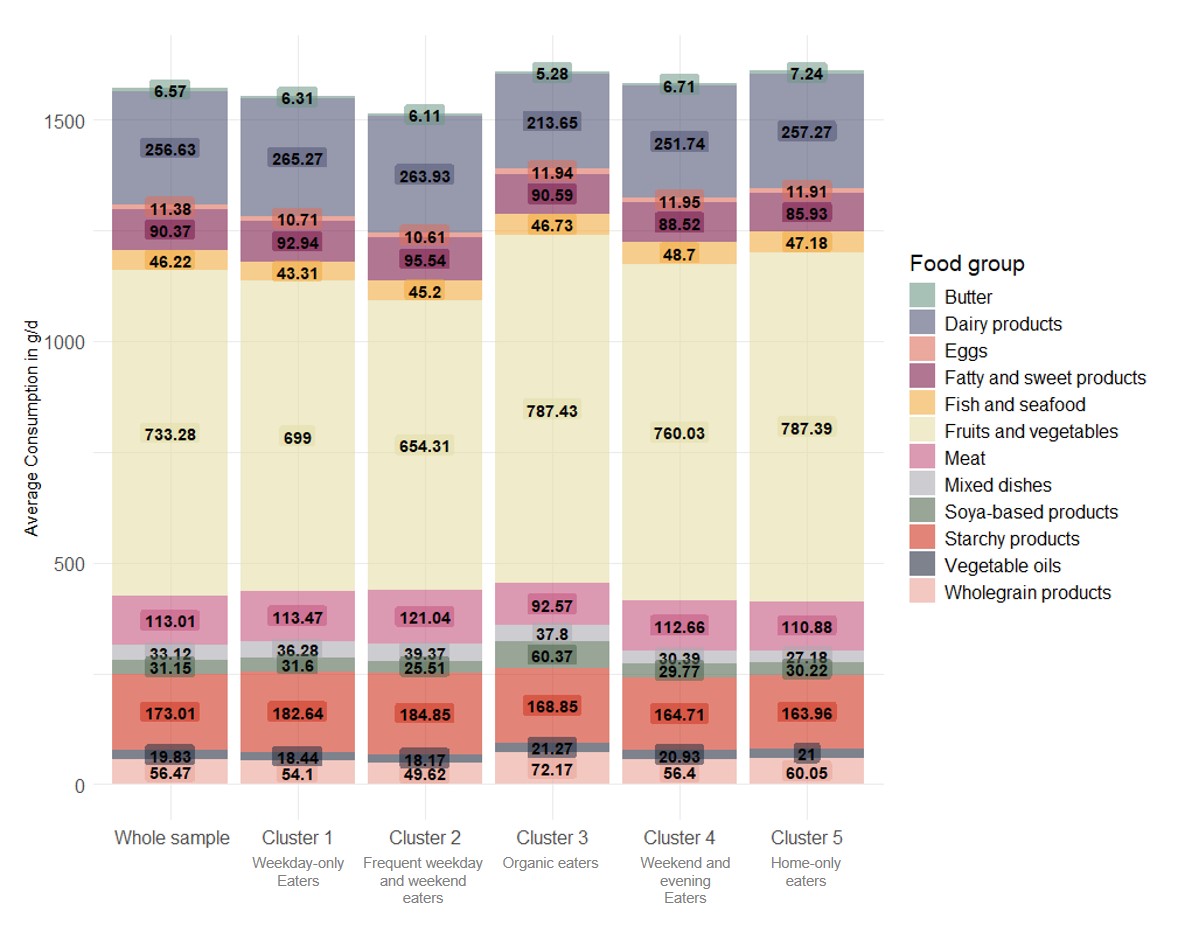
Values are energy-adjusted means of intakes (g/d) derived from ANCOVA. Food groups are constructed as follows: Butter includes all forms of butter; Dairy products include milk, yogurts, fresh cheese, cheese; Eggs include all forms of eggs; Fatty and sweet products include milky desserts, cookies, sweets, croissants, pastries, chocolate, biscuits, ice cream, honey and marmalade, cakes, chips, salted oilseeds, salted biscuits, dressings; Fish includes fish and seafood; Fruits and vegetables include all vegetables, soups, fresh fruits, fruit juice, compote, dried fruits; Meat includes all types of meat (pork, beef, poultry, offal, etc.) and processed meat; Mixed dishes include sandwich, prepared foods such as pizza, hamburger, ravioli, panini, salted pancake, etc.; Soya-based products include tofu, soya-based meat substitute and vegetable patties, soya-based yogurt, soya-based milk; Starchy products include legumes, grains, potatoes, cereals, bread; Vegetable oils include different types of plant-based oils; Wholegrain products include wholegrain pasta, rice, bread. Drinks are excluded.

Supplemental Material References

1. Kesse-Guyot E, Chaltiel D, Fezeu LK, Baudry J, Druesne-Pecollo N, Galan P, et al. Association between adherence to the French dietary guidelines and the risk of type 2 diabetes. Nutrition. 2021 Apr;84:111107.

2. Gavelle E de, Huneau JF, Mariotti F. Patterns of Protein Food Intake Are Associated with Nutrient Adequacy in the General French Adult Population. Nutrients. 2018 Feb 17;10(2):226.

3. Keaver L, Ruan M, Chen F, Du M, Ding C, Wang J, et al. Plant- and animal-based diet quality and mortality among US adults: a cohort study. British Journal of Nutrition. 2021 Jun;125(12):1405–15.

4. EAT. The Planetary Health Diet [Internet]. [cited 2024 Jul 24]. Available from: https://eatforum.org/eat-lancet-commission/the-planetary-health-diet-and-you/

5. Kesse-Guyot E, Rebouillat P, Brunin J, Langevin B, Allès B, Touvier M, et al. Environmental and nutritional analysis of the EAT-Lancet diet at the individual level: insights from the NutriNet-Santé study. Journal of Cleaner Production. 2021 May;296:126555.

6. Francois Husson, Julie Josse, Sebastien Le, Jeremy Mazet. FactoMineR: Multivariate Exploratory Data Analysis and Data Mining [Internet]. 2006 [cited 2024 Jul 24]. p. 2.11. Available from: https://CRAN.R-project.org/package=FactoMineR
